# Management of chronic spontaneous urticaria: a worldwide perspective

**DOI:** 10.1186/s40413-018-0193-4

**Published:** 2018-07-04

**Authors:** Pavel Kolkhir, Dmitry Pogorelov, Razvigor Darlenski, Marco Caminati, Luciana Kase Tanno, Duy Le Pham, Alexei Gonzalez-Estrada, Darío Antolín-Amérigo, Ves Dimov, Karsten Weller, Mario Sánchez-Borges, Ignacio Ansotegui, Marcus Maurer, on behalf of the WAO Junior Members Group

**Affiliations:** 1 Division of Immune-mediated skin diseases, I.M. Sechenov First Moscow State Medical University, 119991, Trubeckaya str., 8/2, Moscow, Russian Federation; 2 Department of Infection and Immunity, Luxembourg Institute of Health, Esch-sur-Alzette, Luxembourg; 3 Department of Dermatology and Venereology, Trakia University, Stara Zagora, Bulgaria; 4 Asthma Center and Allergy Unit, Verona University Hospital, Verona, Italy; 5 Hospital Sírio Libanês and Post-graduation Program in Health Sciences of IAMSPE São Paulo, São Paulo, Brazil; 6 University Hospital of Montpellier, Montpellier and Sorbonne Universités, Paris, France; 7 Faculty of Medicine, University of Medicine and Pharmacy at Ho Chi Minh City, Ho Chi Minh City, Viet Nam; 8 Division of Allergy, Pulmonary and Sleep Medicine, Department of Medicine, Mayo Clinic, Jacksonville, Florida, USA; 9 Servicio de Enfermedades del Sistema Inmune-Alergia, Hospital Universitario Príncipe de Asturias, Madrid, Spain; 10 Departamento de Medicina y Especialidades Médicas, Facultad de Medicina, Universidad de Alcalá, Madrid, Spain; 11 Department of Allergy and Immunology, Cleveland Clinic Florida, Weston, Florida, USA; 12 Department of Dermatology and Allergy, Charité – Universitätsmedizin Berlin, Berlin, Germany; 13 Allergy and Clinical Immunology Department, Centro Medico Docente La Trinidad, Caracas, Venezuela; 14 Department of Allergy and Immunology, Hospital Quirónsalud Bizkaia, Bilbao, Spain

**Keywords:** Chronic spontaneous urticaria, Guidelines, Worldwide, Guideline adherence, Urticaria treatment, Urticaria management, Global survey

## Abstract

**Background:**

The approaches to the diagnosis and treatment of chronic spontaneous urticaria (CSU) differ in various parts of the world. We sought to determine the adherence to international and national urticaria guidelines as well as the motives to deviate from the guidelines among physicians worldwide.

**Methods:**

A web-based questionnaire was created and launched via e-mail by the World Allergy Organization (WAO) to representatives of all WAO Member Societies, the members of the American Academy of Allergy, Asthma & Immunology (AAAAI) and the members of the WAO Junior Members Group (JMG), regardless of the specialty, affiliation, or nationality in March 2017.

**Results:**

We received 1140 completed surveys from participating physicians from 99 countries. Virtually all participants (96%) were aware of at least one urticaria guideline and reported that they follow a guideline. However, one in five physicians who follow a guideline (22%) reported to deviate from it. Reliance on own clinical experience is the most frequent reason for deviation from guidelines or not following them (44%). Young (< 40 years) and less experienced physicians more often follow a guideline and less often deviate than older and experienced ones. Physicians who follow a urticaria guideline showed higher rates of routinely ordering a complete blood count, the erythrocyte sedimentation rate, C-reactive protein, anti-thyroid antibodies, and thyroid-stimulating hormone and of performing the autologous serum skin test as compared to those who do not. Physicians who follow a urticaria guideline showed higher rates of using second generation antihistamines as their first-line treatment of CSU (*p* = 0.001) and more frequently observed higher efficacy of these drugs (or had more confidence that it would work, *p* < 0.019) as compared to those who do not follow the guidelines.

**Conclusions:**

Physicians’ characteristics (e.g. age, clinical experience, and specialty) and country specifics and regional features (e.g. availability of drugs for CSU treatment) importantly influence adherence to urticaria guidelines and CSU patient care and should be addressed in more detail in future research.

**Electronic supplementary material:**

The online version of this article (10.1186/s40413-018-0193-4) contains supplementary material, which is available to authorized users.

## Background

Chronic spontaneous urticaria (CSU) is a mast cell-driven disease that is defined as the occurrence of wheals, angioedema, or both for more than 6 weeks due to known or unknown causes [1]. CSU affects up to 1% of the general population [2, 3]. It exerts a devastating impact on patients’ quality of life [4, 5].

The approaches to the diagnostic workup and treatment of CSU patients differ in various parts of the world, and there are discrepancies between national consensus papers and guidelines and the international EAACI/GA^2^LEN/EDF/WAO guideline [1, 6, 7]. The impact of guidelines on the diagnostic workup and treatment strategy selection in every day clinical practice needs further research. How many physicians know urticaria guidelines? How many physicians use them to guide their clinical practice? What are the reasons for not following the available guidelines? What is the impact of following the guidelines on the quality of care for urticaria patients? These questions need to be addressed on a global level. The answers to these questions can be of significant value in updating and revising the current guidelines and improving patient care.

The World Allergy Organization (WAO) Junior Members Group (JMG) Steering Committee developed a questionnaire to survey the opinions on a whole variety of questions regarding CSU management and the use of guidelines. The questionnaire targeted the WAO members, including, but not limited to the representatives of the constituent national societies of WAO, having the authority to vote on their behalf, the WAO JMG and the American Academy of Allergy, Asthma & Immunology (AAAAI) members. We sought to determine, in physicians from around the world, the knowledge of and adherence to international and national urticaria guidelines as well as the motives to deviate from them.

## Methods

### Study survey

A web-based questionnaire (Additional file 1: Figure S1) was created and circulated among the members of the WAO JMG Steering Committee for revisions (July–September 2016). The protocol was approved by the WAO Executive Committee and Board of Directors (25 October 2016). The questionnaire was created de novo and has not been previously validated. The final version consisted of 24 questions including survey participant demographic information (country of residence, gender, age, specialty, clinical experience and type of practice) and those concerning CSU management (patients’ age, number of CSU patients seen per week, number of CSU patients with angioedema, awareness, adherence and/or deviation of current guidelines, examination of a CSU patient, including general laboratory work-up and targeted search for the cause of the disease, and treatment options used). There were 11 single-choice and 13 multiple-choice questions.

### Recruitment and dissemination

The survey was beta tested and approved by the WAO JMG Steering Committee and WAO leadership before dissemination among participants. It was disseminated via email by the WAO office to representatives of WAO Member Societies as well as members of AAAAI and the WAO JMG in March 2017, with no restrictions applied to the specialty, affiliation, or nationality of the participants. The email contained a link (Internet address) to the online questionnaire that was unique to each participating member. A reminder to participate was sent in April 2017. Participants were given 30 days to reply and were guaranteed complete anonymity.

### Statistical analysis

SPSS v.22 (Armonk, NY: IBM Corp, USA) was used for all analyses. Analyses of the difference in frequencies across groups were performed with the Pearson Chi-squared test and a *p* value ≤0.05 was considered significant.

## Results

### Demographics of study participants

A total of 32,356 individuals from 149 countries were invited to take part in the survey. We received 1140 (3.5%) completed surveys from participating physicians from 99 countries, with most residing in Europe (33.2%) and North America (28.9%) (Table 1). Most of the respondents were allergists/clinical immunologists (88.7%), followed by pediatricians (16.5%), dermatologists (4.5%) and general practitioners (2.2%) (Table 2). One hundred and fifty-seven participants had more than one specialty. The majority of participants were ≥ 40 years old (74.8%) and almost half of respondents had clinical experience of > 19 years (43.9%). Two thirds and half of the participants worked in academic institutions and/or had a private practice. Most participants (88.9%) reported to see primarily outpatients, both adults and children with CSU (53.7%). Only 22% of physicians reported to see ≥10 CSU patients per week.

**Table 1:** A geographical distribution of the respondents participating in the survey (*n* = 1140)

North America (*n* = 330, 28.9%)	Latin America (*n* = 193, 16.9%)	Europe (*n* = 379, 33.2%)	Africa/Middle-East (*n* = 64, 5.6%)	Asia-Pacific (*n* = 174, 15.3%)
Canada	Argentina	Albania	Algeria	Australia
United States	Bolivia	Armenia	Cyprus	Bangladesh
US Virgin Islands	Brazil	Austria	Egypt	Cambodia
	Chile	Azerbaijan	Ethiopia	Hong Kong
	Colombia	Belarus	Iran	India
	Costa Rica	Belgium	Israel	Indonesia
	Cuba	Bulgaria	Kenya	Japan
	Dominican Republic	Croatia	Lebanon	Jordan
	Ecuador	Czech Republic	Oman	Korea
	El Salvador	Denmark	Qatar	Kuwait
	Guatemala	Estonia	South Africa	Malaysia
	Honduras	Finland	Tunisia	Mongolia
	Mexico	France		Nepal
	Panama	Georgia		New Zealand
	Paraguay	Germany		Pakistan
	Peru	Greece		Peoples Republic of China
	Uruguay	Guernsey		Philippines
	Venezuela	Hungary		Saudi Arabia
		Iceland		Singapore
		Ireland		Sri Lanka
		Italy		Taiwan
		Kosovo		Thailand
		Latvia		United Arab Emirates
		Lithuania		Uzbekistan
		Macedonia		Viet Nam
		Moldova		
		Montenegro		
		Netherlands		
		Poland		
		Portugal		
		Romania		
		Russia		
		Serbia		
		Slovakia		
		Slovenia		
		Spain		
		Sweden		
		Switzerland		
		Turkey		
		Ukraine		
		United Kingdom

**Table 2:** Characteristics of survey respondents (*n* = 1140)

Characteristics of respondents		Geographical regions					Total *% (n)*
		NA *% (n/total)*	LA *% (n/total)*	EU *% (n/total)*	AME *% (n/total)*	AP *% (n/total)*	
Specialty^a^ (*n* = 1138)							
Allergists/ Clinical Immunologists		98.2 (324/330)	97.4 (187/192)	83.1 (315/379)	79.7 (51/64)	76.9 (133/173)	88.7 (1010)
Dermatologists		0.3 (1/330)	0.5 (1/192)	9.8 (37/379)	0	6.9 (12/173)	4.5 (51)
Pediatricians		4.2 (14/330)	25.5 (49/192)	17.4 (66/379)	23.4 (15/64)	25.4 (44/173)	16.5 (188)
General Practitioners		0.6 (2/330)	3.1 (6/192)	1.6 (6/379)	6.2 (4/64)	4 (7/173)	2.2 (25)
Gender (*n* = 1117)							
Male		58.5 (189/323)	55.1 (102/185)	43.7 (163/373)	57.1 (36/63)	58.4 (101/173)	53.0 (591)
Female		41.5 (134/323)	44.9 (83/185)	56.3 (210/373)	42.9 (27/63)	41.6 (72/173)	47.0 (526)
Age, years (*n* = 1132)							
< 40		22.9 (75/327)	27.6 (53/192)	28.2 (106/376)	17.2 (11/64)	23.1 (133/173)	25.2 (285)
≥40		77.1 (252/327)	72.4 (139/192)	71.8 (270/376)	82.8 (53/64)	76.9 (133/173)	74.8 (847)
Clinical experience, years (*n* = 1130)							
≤19		50.3 (164/326)	53.9 (104/193)	58.2 (217/373)	60.9 (39/64)	63.2 (110/174)	56.1 (634)
Over 19		49.7 (162/326)	46.1 (89/193)	41.8 (156/373)	39.1 (25/64)	36.8 (64/174)	43.9 (496)
Place of work^a^ (n = 1140)							
Private practice		63.9 (211/330)	82.4 (159/193)	34.6 (131/379)	54.7 (35/64)	46.0 (80/174)	54.0 (616)
University clinic		70.3 (232/330)	68.4 (132/193)	57.5 (218/379)	59.4 (38/64)	71.8 (125/174)	65.3 (745)
Hospital		11.8 (39/330)	39.4 (76/193)	43.3 (164/379)	42.2 (27/64)	58.6 (102/174)	35.8 (408)
Specialized urticaria centre		0.9 (3/330)	4.7 (9/193)	2.9 (11/379)	3.1 (2/64)	0.3 (3/174)	2.4 (28)
Department (n = 1140)							
Outpatients		97.3 (321/330)	89.6 (173/193)	82.3 (312/379)	81.3 (52/64)	89.1 (155/174)	88.9 (1013)
Inpatients		0.9 (3/330)	8.8 (17/193)	12.4 (47/379)	10.9 (7/64)	6.9 (12/174)	7.5 (86)
Outpatients and inpatients		1.8 (6/330)	1.6 (3/193)	5.3 (20/379)	7.8 (5/64)	4.0 (7/174)	3.6 (41)
Age of patients (*n* = 1131)							
Adults		16.5 (54/327)	17.2 (33/192)	42.0 (158/376)	10.9 (7/64)	30.8 (53/172)	27.0 (305)
Children		9.5 (31/327)	16.7 (32/192)	19.4 (73/376)	18.8 (12/64)	27.9 (71/172)	19.3 (219)
Adults and children		74.0 (242/327)	66.1 (127/192)	38.6 (145/376)	70.3 (45/64)	41.3 (48/172)	53.7 (607)
Number of CSU patients per week (*n* = 1127)							
< 10		75.5 (247/327)	78.8 (152/193)	80.7 (302/374)	71.0 (44/62)	78.4 (134/171)	78.0 (879)
≥10		24.5 (80/327)	21.2 (41/193)	19.3 (72/374)	29.0 (18/62)	21.6 (37/171)	22.0 (248)
Patients with angioedema, % from the total number of CSU patients (n = 1131)							
≤20		37.7 (123/326)	58.5 (113/193)	51.7 (195/377)	55.6 (35/63)	66.3 (114/172)	51.3 (580)
> 20		62.3 (203/326)	41.5 (80/193)	48.3 (182/377)	44.4 (28/63)	33.7 (58/172)	48.7 (551)
Adherence to the urticaria guidelines^a^ (*n* = 1126)							
Follow the guidelines	Any of three below	88.0 (286/325)	93.8 (180/192)	97.3 (365/375)	85.7 (54/63)	89.5 (153/171)	92.2 (1038)
	EAACI/WAO/GA^2^LEN/EDF	16.3 (53/325)	78.1 (150/192)	82.4 (309/375)	68.3 (43/63)	63.2 (108/171)	58.9 (663)
	US practice parameters	81.2 (264/325)	32.3 (62/192)	9.1 (34/375)	28.6 (18/63)	29.8 (51/171)	38.1 (429)
	National	7.1 (23/325)	26.0 (50/192)	32.5 (122/375)	14.3 (9/63)	30.4 (52/171)	22.7 (256)
	Follow any but deviate	40.0 (80/200)	28.1 (25/89)	49.4 (79/160)	58.1 (18/31)	53.8 (49/91)	22.3 (251)
Do not follow		12.0 (39/325)	6.3 (12/192)	2.7 (10/375)	14.3 (9/63)	10.5 (18/171)	7.8 (88)

*AME* Africa/Middle-East, *AP* Asia-Pacific, *EU* Europe, *LA* Latin America, *NA* North America. ^a^respondents could choose more than one option

### More than 90% of physicians follow the urticaria guidelines, but almost one-fourth of them deviate

**Fig. 1:**
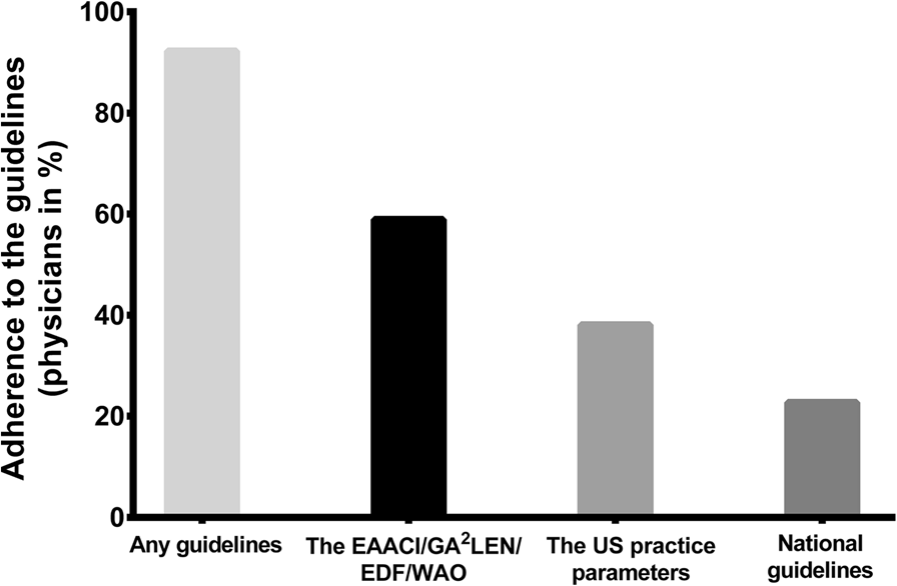
Adherence to the urticaria guidelines. Physicians were asked if they know and follow the current guidelines for management of urticaria. Results are expressed as percentage of participants who chose the corresponding guidelines (one respondent could choose several answers)

Virtually all participants (1086 of 1126, 96%) were aware of one or more urticaria guidelines, and almost all of them reported to follow a guideline (*n* = 1038 of 1086, 96%) (Fig. 1). The most widely used guideline was the international EAACI/GA^2^LEN/EDF/WAO urticaria guideline [1] (58.9%), followed by the American AAAAI/ACAAI Joint Task Force practice parameters for the diagnosis and management of acute and chronic urticaria [7] (38.1%) and national guidelines (22.7%). Expectedly, the US Joint Task Force practice parameters are used more often in North America and the EAACI/GA^2^LEN/EDF/WAO urticaria guidelines are more known in other countries of the world. One in five physicians who follow a guideline (22%) reported to deviate from it.

### Reliance on own clinical experience is the most frequent reason for deviation from the guidelines or not following them

Of the 339 (30%) physicians who do not follow a guideline or follow a guideline but deviate from it, 149 (43.9%) reported that they rely more on their own clinical experience (Table 3). It was the most frequent reason in all regions of the world except for Latin America, where the most common reason, provided by 14.3% of respondents, was that some of the guidelines’ recommendations cannot be implemented in their country of residence. The second most prevalent reason for deviating from guideline recommendations (29.8%) was that the approach to CSU management recommended by the guidelines was seen as overly simplified and not reflecting the complexity of the disease. The least frequent reasons were the discrepancy and/or disagreement between the guidelines (3.8%) and a negative experience with following the guidelines in clinical practice (3.2%). Nine percent of participants did not agree with the guidelines’ recommendations and/or conclusions.

**Table 3:** Reasons why physicians don’t follow or deviate from the guidelines

Reasons	Geographical regions					Total *n* = 339^a^ % (n)
	NA *n* = 236^a^ *% (n)*	LA *n* = 98^a^ *% (n)*	EU *n* = 167^a^ *% (n)*	AME *n* = 38^a^ *% (n)*	AP *n* = 104^a^ *% (n)*	
The guidelines do not undergo revision frequently enough	4.2 (10)	4.1 (4)	3.0 (5)	5.3 (2)	6.7 (7)	8.2 (28)
I rely more on my own clinical experience	27.1 (64)	10.2 (10)	20.4 (34)	34.2 (13)	26.9 (28)	43.9 (149)
I do not agree with the guidelines’ recommendations and/or conclusions	7.2 (17)	3.1 (3)	4.2 (7)	2.6 (1)	3.8 (4)	9.4 (32)
Some of the recommendations are unclear to me and require further details	3.4 (8)	1.0 (1)	8.4 (14)	2.6 (1)	11.5 (12)	10.6 (36)
Some of the guidelines’ recommendations cannot be implemented in my country of residence	0.8 (2)	14.3 (14)	12.0 (20)	26.3 (10)	22.1 (23)	20.3 (69)
I had a negative experience with following the guidelines in my clinical practice	1.7 (4)	0 (0)	3.6 (6)	2.6 (1)	0 (0)	3.2 (11)
Overly simplified approach to CSU management recommended by the guidelines that does not reflect the complexity of the disease	22.9 (54)	7.1 (7)	15.6 (26)	10.5 (4)	9.6 (10)	29.8 (101)
The discrepancy and/or disagreement between the guidelines	3.0 (7)	2.0 (2)	1.8 (3)	2.6 (1)	0 (0)	3.8 (13)

*AME* Africa/Middle-East, *AP* Asia-Pacific, *EU* Europe, *LA* Latin America, *NA* North America. ^a^the total number of respondents was 339. However, there was overlapping in the data analysis because respondents could choose more than one option

### Young and less experienced physicians more often follow a guideline and less often deviate than older and experienced ones

Physicians who are less than 40 years of age more often reported that they adhere to urticaria guidelines and less often deviate as compared to responding physicians of ≥40 years of age (*p* = 0.001 and *p* = 0.023, respectively). Responding physicians with clinical experience of > 19 years statistically more often deviate from the guidelines and less frequently follow them as compared to responding physicians with clinical experience of 19 years or less (*p* = 0.025 and *p* < 0.001, respectively) (Tables 4 and 5).

**Table 4:** Factors linked to adherence to the urticaria guidelines

Factors	% (n) of physicians, who follow the guidelines	% (n) of physicians, who don’t follow the guidelines	X^2^	p
Age, years				
< 40 (*n* = 283)	96.9 (274)	3.1 (9)	11.152	**0.001**
≥40 (*n* = 836)	90.7 (758)	9.3 (78)		
Clinical experience, years				
≤19 years (*n* = 630)	95.9 (604)	4.1 (26)	28.017	**< 0.001**
Over 19 years (*n* = 487)	87.3 (425)	12.7 (62)		
Department^a^				
Outpatients (*n* = 1006)	92.0 (926)	8.0 (80/85)	0.431	0.511
Inpatients (*n* = 84)	94.0 (79)	6.0 (5)		
Age of patients^a^				
Adults (*n* = 300)	92.3 (277)	7.7 (23)	0.029	0.865
Children (n = 193)	92.7 (179)	7.3 (14)		
CSU patients per week				
< 10 (*n* = 870)	92.2 (802)	7.8 (68)	0.076	0.783
≥10 (*n* = 247)	92.7 (229)	7.3 (18)		

^a^Only in physicians who chose one of the options

Values marked in bold indicate a statistically significant difference (*p* < 0.05)

**Table 5:** Factors linked to the deviation from the urticaria guidelines

Factors	% (*n*) of physicians, who deviate from the guidelines	% (*n*) of physicians, who don’t deviate from the guidelines	X^2^	*p*
Age, years				
< 40 (*n* = 140)	35.7 (50)	64.3 (90)	5.194	**0.023**
≥40 (*n* = 428)	46.7 (200)	53.3 (228)		
Clinical experience, years				
≤19 years (*n* = 323)	39.9 (129)	60.1 (194)	5.049	**0.025**
Over 19 years (*n* = 245)	49.4 (121)	50.6 (124)		
Patients^a^				
Outpatients (*n* = 510)	44.5 (227)	55.5 (283)	0.029	0.865
Inpatients (n = 44)	43.2 (19)	56.8 (25)		
Age of patients^a^				
Adults (*n* = 150)	55.3 (83)	44.7 (67)	7.712	**0.005**
Children (*n* = 92)	37.0 (34)	63.0 (58)		
CSU patients per week				
< 10 (*n* = 441)	44.0 (194)	50.0 (247)	0.002	0.961
≥10 (*n* = 128)	43.7 (56)	56.3 (72)		

^a^Only in physicians who chose one of the options

Values marked in bold indicate a statistically significant difference (*p* < 0.05)

### Physicians who follow a urticaria guideline more often perform diagnostic tests

Physicians who follow a urticaria guideline showed higher rates of routinely ordering a complete blood count (CBC), the erythrocyte sedimentation rate (ESR), C-reactive protein (CRP), anti-thyroid antibodies, and thyroid-stimulating hormone (TSH) and of performing the autologous serum skin test (ASST) as compared to those who do not (Table 6). CSU due to unknown causes was reported to be much more common than CSU due to known causes, in all regions of the world (90 vs 10%). Autoimmunity was the most common identifiable cause of CSU (51.9%) and malignancy was the least common identifiable cause of CSU (4.5%). Food intolerance was a less frequent cause of CSU in North America (8.0%) as compared to other regions of the world (> 16.0%) (Table 7).

**Table 6:** Differences in the approach to the management of CSU in respondents who do and do not follow the guidelines

Test	Compared groups	*n*	% (n) of physicians, who follow the guidelines	% (n) of physicians, who don’t follow the guidelines	X^2^	*p*
CBC	Order	854	79.2 (802)	60.5 (52)	16.011	**< 0.001**
	No	245	20.8 (211)	39.5 (34)		
ESR	Order	602	55.9 (566)	41.9 (36)	6.284	**0.012**
	No	497	44.1 (447)	58.1 (50)		
CRP	Order	527	49.0 (496)	36.0 (31)	5.299	**0.021**
	No	572	51.0 (517)	64.0 (55)		
Anti-TG/TPO	Order	559	51.9 (526)	38.4 (33)	5.826	**0.016**
	No	540	48.1 (487)	61.6 (53)		
TSH	Order	543	50.8 (515)	32.6 (28)	10.598	**0.001**
	No	556	49.2 (498)	67.4 (58)		
Total IgE	Order	481	56.1 (445)	58.1 (36)	0.138	0.710
	No	618	43.9 (568)	41.9 (50)		
ECP	Order	51	4.7 (48)	3.5 (3)	0.280	0.597
	No	1048	95.3 (965)	96.5 (83)		
D-dimer	Order	54	5.2 (53)	1.2 (1)	2.809	0.094
	No	1045	94.8 (960)	98.8 (85)		
Skin prick tests	Order	308	71.4 (290)	79.1 (18)	2.329	0.127
	No	791	28.6 (723)	20.9 (68)		
Allergen-specific IgE	Order	286	26.5 (268)	20.9 (18)	1.257	0.262
	No	813	73.5 (745)	79.1 (68)		
ANA	Order	407	37.7 (382)	29.1 (25)	2.538	0.111
	No	692	62.3 (631)	70.9 (61)		
Tryptase	Order	162	84.9 (153)	89.5 (9)	1.357	0.244
	No	937	15.1 (860)	10.5 (77)		
ASST	Order	186	17.7 (179)	8.1 (7)	5.121	**0.024**
	No	913	82.3 (834)	91.9 (79)		
Search for chronic infections	Perform	364	66.2 (342)	74.4 (22)	2.394	0.122
	No	735	33.8 (671)	25.6 (64)		
Do not order any tests		182	15.4 (156)	30.2 (26)	12.621	**< 0.001**
Order at least 1 test		917	84.6 (857)	69.8 (60)		

*CBC* complete blood count, *ESR* erythrocyte sedimentation rate, *CRP* C-reactive protein, *TG/TPO* thyroglobulin/thyroperoxidase, *TSH* thyroid-stimulating hormone, *ECP* eosinophil cationic protein, *ANA* antinuclear antibodies, *ASST* autologous serum skin test

Values marked in bold indicate a statistically significant difference (*p* < 0.05)

**Table 7:** Number of respondents from different regions of the world who find these causes of CSU as most common (*n* = 1098)^a^

CSU causes	Geographical regions					Total % (n/total)
	NA *% (n/total)*	LA *% (n/total)*	EU *% (n/total)*	AME *% (n/total)*	AP *% (n/total)*	
Idiopathic CSU	97.5 (315/323)	80.6 (150/186)	88 (323/367)	93.3 (56/60)	89.5 (145/162)	90.1 (989/1098)
Type-I-allergy	27.5 (77/280)	27.1 (46/170)	17.9 (60/336)	19.6 (11/56)	39.6 (59/149)	25.5 (253/991)
Autoimmune CSU	64.4 (201/312)	44.8 (81/181)	46.8 (166/355)	32.8 (19/58)	54.1 (80/148)	51.9 (547/1054)
Systemic disorders	26.7 (79/296)	29.1 (50/172)	18.2 (62/340)	12.3 (7/57)	20.8 (31/149)	22.6 (229/1014)
Malignancy	2.4 (7/288)	6.1 (10/165)	6.6 (22/334)	0 (0/56)	3.6 (5/139)	4.5 (44/982)
Chronic infection	11.7 (34/290)	32.8 (58/177)	27.4 (95/347)	13.6 (8/59)	22.3 (33/148)	22.3 (228/1021)
Food intolerance	8.0 (23/289)	19.7 (35/178)	16.3 (56/343)	22.4 (13/58)	26.6 (41/154)	16.4 (168/1022)

*AME* Africa/Middle-East, *AP* Asia-Pacific, *EU* Europe, *LA* Latin America, *NA* North America. ^a^the respondents could choose more than one answer

### Adherence to urticaria guidelines is associated with more frequent administration and confidence in higher efficacy of second-generation antihistamines

Updosing of second-generation H1-antihistamines (sgAHs, 97%) and omalizumab (96%) were reported to be the most effective treatment options in all regions of the world. Dapsone, montelukast and H2-antihistamines were considered effective drugs for treatment of CSU worldwide only by 17, 17 and 15% physicians, respectively. Less respondents from North America as compared to other regions of the world reported that sgAHs at standard dose and montelukast are highly effective (48% vs 60–76 and 9% vs 13–35%, respectively). Vice versa, more physicians from North America as compared to other countries reported that tricyclic antidepressants are highly effective (52% vs 15–33%). Physicians who follow a urticaria guideline showed higher rates of sgAHs administration as a first-line treatment of CSU (*p* = 0.001) and more frequently observed higher efficacy of treatment (or had more confidence that it would work, *p* < 0.019) as compared to those who do not follow the guidelines (Table 8). Guideline followers more frequently use higher than standard-dosed sgAHs and omalizumab as a second and third line treatment, respectively, and less frequently administer first generation antihistamines, tricyclic antidepressants and systemic corticosteroids in comparison to physicians who do not follow a urticaria guideline (Tables 9 and 10).

**Table 8:** Differences in the approach to a first line treatment of CSU in physicians who do and do not follow the guidelines

Treatment	Compared groups	*n*	% (*n*) of physicians, who follow the guidelines	% (*n*) of physicians, who don’t follow the guidelines	X^2^	*p*
First-generation H1-antihistamines	Administer	173	15.4 (160)	14.8 (13)	0.026	0.873
	No	953	84.6 (878)	85.2 (75)		
Second-generation H1-antihistamines at standard dose	Administer	704	64 (664)	45.5 (40)	11.868	**0.001**
	No	422	36 (374)	54.5 (48)		
Updosed second-generation H1-antihistamines	Administer	540	47.8 (496)	50.0 (44)	0.160	0.690
	No	586	52.2 (542)	50.0 (44)		
H2-antihistamines (e.g. famotidine or ranitidine)	Administer	224	19.7 (204)	22.7 (20)	0.481	0.488
	No	902	80.3 (834)	77.3 (68)		
Ciclosporin	Administer	15	1.4 (15)	0	1.289	0.256
	No	1111	98.6 (1023)	100 (88)		
Omalizumab	Administer	32	3.0 (31)	1.1 (1)	1.006	0.316
	No	1094	97.0 (1007)	98.9 (87)		
Montelukast	Administer	150	13.2 (137)	14.8 (13)	0.174	0.676
	No	976	86.8 (901)	85.2 (75)		
Dapsone	Administer	7	0.6 (6)	1.1 (1)	0.409	0.522
	No	1119	99.4 (1032)	98.9 (87)		
Systemic corticosteroids (for less than 10 days)	Administer	215	18.7 (194)	23.9 (21)	1.406	0.236
	No	911	81.3 (844)	76.1 (67)		
Systemic corticosteroids (for more than 10 days in a row)	Administer	16	1.5 (16)	0	1.376	0.241
	No	1110	98.5 (1022)	100 (88)		
Tricyclic antidepressants (e.g. doxepin)	Administer	35	3.0 (31)	4.5 (4)	0.655	0.418
	No	1091	97.0 (1007)	95.5 (84)		

Values marked in bold indicate a statistically significant difference (*p* < 0.05)

**Table 9:** Differences in the approach to a second line treatment of CSU in physicians who do and do not follow the guidelines

Treatment	Compared groups	*n*	% (*n*) of physicians, who follow the guidelines	% (*n*) of physicians, who don’t follow the guidelines	X^2^	*p*
First-generation H1-antihistamines	Administer	156	13.1 (136)	22.7 (20)	6.297	**0.012**
	No	970	86.9 (902)	77.3 (68)		
Second-generation H1-antihistamines at standard dose	Administer	127	11.3 (117)	11.4 (10)	0.001	0.979
	No	999	88.7 (921)	88.6 (78)		
Updosed second-generation H1-antihistamines	Administer	651	58.8 (610)	46.6 (41)	4.931	**0.026**
	No	475	41.2 (428)	53.4 (47)		
H2-antihistamines (e.g. famotidine or ranitidine)	Administer	308	27.1 (281)	30.7 (27)	0.532	0.466
	No	818	72.9 (757)	69.3 (61)		
Ciclosporin	Administer	76	6.8 (71)	5.7 (5)	0.173	0.678
	No	1050	93.2 (967)	94.3 (83)		
Omalizumab	Administer	163	14.5 (150)	14.8 (13)	0.007	0.934
	No	963	85.5 (888)	85.2 (75)		
Montelukast	Administer	391	35.5 (368)	26.1 (23)	3.106	0.078
	No	735	64.5 (670)	73.9 (65)		
Dapsone	Administer	39	3.2 (33)	6.8 (6)	3.213	0.073
	No	1087	96.8 (1005)	93.2 (82)		
Systemic corticosteroids (for less than 10 days)	Administer	265	23.8 (247)	20.5 (18)	0.503	0.478
	No	861	76.2 (791)	79.5 (70)		
Systemic corticosteroids (for more than 10 days in a row)	Administer	89	7.4 (77)	13.6 (12)	4.309	**0.038**
	No	1037	92.6 (961)	86.4 (76)		
Tricyclic antidepressants (e.g. doxepin)	Administer	137	11.6 (120)	19.3 (17)	4.568	**0.033**
	No	989	88.4 (918)	80.7 (71)		

Values marked in bold indicate a statistically significant difference (*p* < 0.05)

**Table 10:** Differences in the approach to a third line treatment of CSU in physicians who do and do not follow the guidelines

Treatment	Compared groups	*n*	% (*n*) of physicians, who follow the guidelines	% (*n*) of physicians, who don’t follow the guidelines	X^2^	*p*
First-generation H1-antihistamines	Administer	93	7.7 (80)	14.8 (13)	5.345	**0.021**
	No	1033	92.3 (958)	85.2 (75)		
Second-generation H1-antihistamines at standard dose	Administer	78	6.9 (72)	6.8 (6)	0.002	0.967
	No	1048	93.1 (966)	93.2 (82)		
Updosed second-generation H1-antihistamines	Administer	283	25.4 (264)	21.6 (19)	0.637	0.425
	No	843	74.6 (774)	78.4 (69)		
H2-antihistamines (e.g. famotidine or ranitidine)	Administer	206	18.3 (190)	18.2 (16)	0.001	0.977
	No	920	81.7 (848)	81.8 (72)		
Ciclosporin	Administer	254	23.1 (240)	15.9 (14)	2.416	0.120
	No	872	76.9 (798)	84.1 (74)		
Omalizumab	Administer	570	51.8 (538)	36.4 (32)	7.764	**0.005**
	No	556	48.2 (500)	63.6 (56)		
Montelukast	Administer	319	28.9 (300)	21.6 (19)	2.135	0.144
	No	807	71.1 (738)	78.4 (69)		
Dapsone	Administer	91	7.8 (81)	11.4 (10)	1.384	0.239
	No	1035	92.2 (957)	88.6 (78)		
Systemic corticosteroids (for less than 10 days)	Administer	227	20.1 (209)	20.5 (18)	0.005	0.943
	No	899	79.9 (829)	79.5 (70)		
Systemic corticosteroids (for more than 10 days in a row)	Administer	146	12.7 (132)	15.9 (14)	0.733	0.392
	No	980	87.3 (906)	84.1 (74)		
Tricyclic antidepressants (e.g. doxepin)	Administer	134	11.7 (121)	14.8 (13)	0.751	0.386
	No	992	88.3 (917)	85.2 (75)		

Values marked in bold indicate a statistically significant difference (*p* < 0.05)

## Discussion

Several guidelines, consensus papers, and practice parameters have been developed for the management of chronic urticaria. Some studies have explored, on the national level, if physicians know these guidelines and implement them in their actual clinical practice [8, 9]. To our knowledge, our study is the first global report of how physicians approach CSU.

### Most physicians know and use urticaria guidelines in their clinical practice

More than 90% of respondents stated to be aware and follow urticaria guidelines. However, there is inconsistency between our study and other studies. For example, most respondents from Latin America in our study (94%) followed any urticaria guideline with 78% followed the EAACI/GA^2^LEN/EDF/WAO urticaria guideline. In contrast, only 79 of 421 (19%) physicians from Ecuador reported to know the EAACI/GA^2^LEN/EDF/WAO urticaria guideline, but more than half of them (67%) were dermatologists and allergists [8]. In German-wide study, only one-third of all physicians participating in the survey were familiar with the EAACI/GA^2^LEN/EDF/WAO urticaria guideline [9]. In Italy, 56% of specialists knew the CSU guidelines and only 27% used them regularly [10]. The high rates of adherence to urticaria guidelines in our study can be explained by increase in guidelines awareness worldwide over time and the fact that most of the participants were allergists/clinical immunologists (88%).

### Factors associated with adherence to guidelines

Young (< 40 years) and less experienced physicians (≤19 years in practice) more often follow guidelines and less often deviate from them than their older and more experienced colleagues. A similar tendency has been observed for other diseases, where low adherence rates to guidelines were also showed to be linked with the advanced age of the physicians [11–13]. For example, old age, male sex, and incomplete residency training were associated with disagreement with clinical practice guidelines for cancer screening [13]. In contrast, compared with physicians ≥50 years, younger physicians (< 50 years) reported a lower level of awareness of cholesterol guidelines [14].

We did not compare the adherence to urticaria guidelines between respondents of different specialties because most physicians in our study were allergists and many of them had several specialties. However, in previous studies the level of knowledge was highest for allergists and/or dermatologists [8, 9], and these physicians have significantly higher expertise in caring for patients with urticaria than other specialists [15]. An observational study from the UK showed that allergists follow the urticaria guidelines more regularly and consistently compared to dermatologists [16]. The results of this study should be evaluated with caution because of the fact that Allergology is recognized as a specialty in some countries (for example, in Russia) or as a subspecialty in others (for example, in Germany).

### Impact of following the guidelines on the quality of care for CSU patients

According to the EAACI/GA^2^LEN/EDF/WAO guideline, only differential blood count and CRP or ESR are recommended as routine diagnostic tests for CSU patients [1]. The US practice parameters recommend limited laboratory testing including a CBC with differential, ESR and/or CRP, liver enzymes, and TSH measurement [7]. Expectedly, these diagnostic tests were performed more frequently by physicians who follow a urticaria guideline in our and other studies [8, 9].

Additional tests are indicated as an extended diagnostic program for identification of underlying causes or eliciting factors and for ruling out possible differential diagnoses if suggested based on history only [1]. For example, allergy is regarded as a very rare cause of CSU [1], and allergy testing is usually not cost-effective and does not lead to improved patient care outcomes [7]. However, some physicians reported to determine total serum IgE (43.8%) and to perform allergy skin prick testing in patients with CSU (28.0%). In a cross-sectional study from Latin America, total serum IgE was the most common diagnostic test (83.5%) [8]. Interestingly, 5–15% of respondents perform other less useful diagnostic tests, e.g. ECP and tryptase, in patients with CSU.

Idiopathic CSU was reported to be the most common type of CSU; this is in the line with other studies [9, 17]. In one study, allergists and dermatologists more frequently searched for CSU etiology as compared to general practitioners [8] in contrast to the results of other study [9]. Although IgE-mediated allergy is a rare cause of CSU [1, 18], IgE-mediated allergy is considered to be a common cause of CSU by 26% of respondents.

Up to 50% of CSU patients can have circulating functional IgG autoantibodies against IgE and high-affinity IgE receptors on mast cells and basophils [19]. Half of respondents reported autoimmune CSU as the most common cause of CSU and 16.9% of physicians (10.1–13.5% in other studies [8, 9]) carried out ASST as a screening method for the detection of autoantibodies [1]. ASST was applied more often by physicians who were aware of and/or follow the guidelines in our and another study [9], but not in all [8].

There is a universal agreement among urticaria guidelines [1, 7, 20] that second generation antihistamines (sgAHs) at a standard dose should be the first line therapy, which is effective in improving symptoms in about 40% of CSU patients [21]. Guideline followers, quite expectedly, use sgAHs at a standard dose as a first line therapy more frequently than non-followers, while the administration of other drugs was not different between the two groups. This has been proved in early national cross-sectional studies where sgAHs taken regularly were the most common drugs prescribed [10, 22]. It is consistent with the finding that more guidelines followers (67.4%) than non-followers (50%) feel that sgAHs are highly effective in CSU treatment.

As a second line therapy, the EAACI/GA^2^LEN/EDF/WAO urticaria guideline recommends the use of sgAHs in higher doses up to four times the standard dose. Physicians who use urticaria guidelines more frequently selected up-dosing for a second-line treatment in our and other studies [9, 10].

For non-respondents to sgAHs up-dosing, the EAACI/GA^2^LEN/EDF/WAO guideline recommends omalizumab, ciclosporin (step 4 in the US practice parameters) or montelukast (step 2 in the US practice parameters) as a third line treatment option [1, 7]. Guideline followers in our and one other study [8] more frequently used omalizumab as a third line treatment in comparison to physicians who do not follow a urticaria guideline.

Our and early studies [9] showed that physicians who are familiar with the guidelines are less likely to use first generation antihistamines as a second and/or third line treatment and systemic steroids (for more than 10 days in a row) as a second line therapy, indicating that guideline recommendations may improve the quality of care [9].

The treatment of CSU can depend on physician’s specialty. For example, Cherrez et al. showed that allergists and dermatologists in Ecuador prescribed significantly more sgAHs (regular doses) as compared to general practitioners [8].

### Reasons for not following or deviation from the available urticaria guidelines

Almost one-third of physicians do not follow a guideline or deviate from it. The most frequent reasons given were reliance on their own clinical experience (44%) and an overly simplified approach to CSU management recommended by the guidelines (30%). Moreover, many physicians, especially those of 40 years or older and with clinical experience of > 19 years, follow guidelines but can deviate from them in some cases, e.g. in difficult-to-treat CSU. This may point to a need to better communicate to physicians, especially experienced physicians, the benefits of guideline adherence and to better engage them in the guideline development and review process. Also, more efforts appear to be needed to improve physician “buy-in” to guidelines by allowing for sufficient flexibility and by educating them that guidelines are meant to complement, rather than substitute for, clinical judgement.

One-fifth of physicians reported that some of the guidelines’ recommendations cannot be implemented in physician’s country of residency. It suggests that economic considerations are an important and often decisive factor influencing the choice of a treatment strategy. For example, omalizumab is unavailable in some countries or its cost is too high and health insurance programs do not cover it (for example, in Russia or Latin America [23]). Systemic steroids and first generation antihistamines are cheaper than sgAHs (for example, in Ecuador [8]) and this can prompt a physician’s decision to prescribe them. The cost-effectiveness of the treatment for CSU, especially in the developing and low-income countries, should be further investigated in future studies.

The EAACI/GA^2^LEN/EDF/WAO guideline is revised every four years by a global panel of well-known experts in the field. Interestingly, 8–10% of respondents did not agree with guidelines’ recommendations and conclusions or found guideline recommendations unclear or outdated. Again, this calls for the consideration of improvements in the development of guideline updates and revisions.

The recommendations given by all of urticaria guidelines are similar, although some differences exist. For example, in contrast to the EAACI/GA^2^LEN/EDF/WAO guideline US practice parameters recommend H2-antagonists and first generation antihistamines for treatment of urticaria as a second or third line therapy, respectively [24]. Only 4% of respondents named the discrepancy and/or disagreement between the guidelines as a reason not to follow them.

Taken together, reliance on own clinical experience, especially in older physicians, rather than economic reasons or unavailability of drugs, appears to be the most frequent reason for deviation from or not following the guidelines. This observation offers the opportunity for a debate on medicine based on experience and evidence-based medicine and highlights the need for continuous medical education for healthcare providers.

### Limitations

The main limitations of our study are the bias of participant selection, the use of an online non-validated questionnaire and a low response rate (3.5%). The fact that most participants in our study were allergists, whereas CSU is often managed by dermatologists and general practitioners, could explain some differences between our findings and those from other studies [8, 9]. There is limited information in regards to CSU management in Africa/Middle-East (only 64 questionnaires were filled out). The most recent EAACI guideline [18] appeared after we performed our study agreeing on our observations.

## Conclusion

The results of our study indicate that urticaria guideline recommendations contribute to a higher quality of patient care. Most physicians worldwide follow a guideline, however, one in five deviates from them. We speculate there are three major reasons for deviation that should be addressed in future research. Firstly, older physicians may be prone to disproportionate reliance on their clinical experience and unable to fully incorporate rapidly emerging evidence-based approaches in their routine clinical practice, which highlights the need for continuous medical education for healthcare providers regardless of their age group or occupying position. Secondly, the quality of CSU patient care may be, to a large degree, compromised by the financial constraints and insufficient level of training of the treating physicians in developing countries. It warrants more research into pharmacoeconomics and sustainability of up-to-date CSU treatments and further propagation of new knowledge about CSU etiopathogenesis and treatment among practicing physicians of different specialties and healthcare authorities in different countries. Finally, urticaria guidelines themselves can be a cause for suboptimal patient care (for example, unclear recommendations and discrepancies between the guidelines). Thus, on the one hand, urticaria guidelines should be flexible enough to allow a physician to tailor the treatment to the unique profile of each patient and circumstances specific to their country of residence; on the other hand, further standardization and dissemination of guidelines can increase adherence among physicians worldwide and result in better patient care.

## Additional file

Additional file 1: The web-based questionnaire. (PDF 180 kb)

## Abbreviations

AAAAI: American Academy of Allergy, Asthma & Immunology
ASST: Autologous serum skin test
CBC: Complete blood count
CRP: C-reactive protein
CSU: Chronic spontaneous urticaria
ESR: Erythrocyte sedimentation rate
JMG: Junior Member Group
sgAHs: Second generation antihistamines
TSH: Thyroid-stimulating hormone
WAO: World Allergy Organization
