# Supplementary material for: Management of chronic spontaneous urticaria: a worldwide perspective
Source: World Allergy Organ J. 2018 Jul 4;11(1):14. doi: 10.1186/s40413-018-0193-4 (PMC6030778; doi:10.1186/s40413-018-0193-4)
Supplement: Supplementary file 1 — The web-based questionnaire. (PDF 180 kb) [file 40413_2018_193_MOESM1_ESM.pdf]

Survey on chronic spontaneous urticaria: a worldwide perspective

1 Your country of residence:

- ◯ Afghanistan
- ◯ Algeria
- ◯ American Samoa
- ◯ Andorra
- ◯ Angola
- ◯ Antigua and Barbuda
- ◯ Argentina
- ◯ Armenia
- ◯ Australia
- ◯ Austria
- ◯ Azerbaijan
- ◯ Bahamas
- ◯ Bahrain
- ◯ Bangladesh
- ◯ Barbados
- ◯ Belarus
- ◯ Belgium
- ◯ Belize
- ◯ Benin
- ◯ Bermuda
- ◯ Bhutan
- ◯ Bolivia
- ◯ Bosnia and Herzegovina
- ◯ Botswana
- ◯ Brazil
- ◯ Brunei
- ◯ Bulgaria
- ◯ Burkina Faso
- ◯ Burundi
- ◯ Cambodia
- ◯ Cameroon
- ◯ Canada
- ◯ Cape Verde
- ◯ Cayman Islands
- ◯ Central African Republic
- ◯ Chad
- ◯ Chile
- ◯ China
- ◯ Colombia
- ◯ Comoros
- ◯ Congo, Democratic Republic of the
- ◯ Congo, Republic of the
- ◯ Costa Rica
- ◯ Côte d'Ivoire
- ◯ Croatia
- ◯ Cuba
- ◯ Cyprus
- ◯ Czech Republic
- ◯ Denmark
- ◯ Djibouti
- ◯ Dominica
- ◯ Dominican Republic
- ◯ East Timor
- ◯ Ecuador
- ◯ Egypt
- ◯ El Salvador
- ◯ Equatorial Guinea
- ◯ Eritrea
- ◯ Estonia
- ◯ Ethiopia
- ◯ Faroe Islands
- ◯ Fiji
- ◯ Finland
- ◯ France
- ◯ French Polynesia
- ◯ Gabon
- ◯ Gambia
- ◯ Georgia
- ◯ Germany
- ◯ Ghana
- ◯ Greece
- ◯ Greenland
- ◯ Grenada
- ◯ Guam
- ◯ Guatemala
- ◯ Guinea
- ◯ Guinea-Bissau
- ◯ Guyana
- ◯ Haiti
- ◯ Honduras
- ◯ Hong Kong
- ◯ Hungary
- ◯ Iceland
- ◯ India
- ◯ Indonesia
- ◯ Iran
- ◯ Iraq
- ◯ Ireland
- ◯ Israel
- ◯ Italy
- ◯ Jamaica
- ◯ Japan
- ◯ Jordan
- ◯ Kazakhstan
- ◯ Kenya
- ◯ Kiribati
- ◯ North Korea
- ◯ South Korea
- ◯ Kosovo
- ◯ Kuwait
- ◯ Kyrgyzstan
- ◯ Laos
- ◯ Latvia
- ◯ Lebanon
- ◯ Lesotho
- ◯ Liberia
- ◯ Libya
- ◯ Liechtenstein
- ◯ Lithuania
- ◯ Luxembourg
- ◯ Macedonia
- ◯ Madagascar
- ◯ Malawi
- ◯ Malaysia
- ◯ Maldives
- ◯ Mali
- ◯ Malta
- ◯ Marshall Islands
- ◯ Mauritania
- ◯ Mauritius
- ◯ Mexico
- ◯ Micronesia
- ◯ Moldova
- ◯ Monaco
- ◯ Mongolia
- ◯ Montenegro
- ◯ Morocco
- ◯ Mozambique
- ◯ Myanmar
- ◯ Namibia
- ◯ Nauru
- ◯ Nepal
- ◯ Netherlands
- ◯ New Zealand
- ◯ Nicaragua
- ◯ Niger
- ◯ Nigeria

○ Northern Mariana Islands
○ Norway
○ Oman
○ Pakistan
○ Palau
○ Palestine, State of
○ Panama
○ Papua New Guinea
○ Paraguay
○ Peru
○ Philippines
○ Poland
○ Portugal
○ Puerto Rico
○ Qatar
○ Romania
○ Russia
○ Rwanda
○ Saint Kitts and Nevis
○ Saint Lucia
○ Saint Vincent and the Grenadines
○ Samoa
○ San Marino
○ Sao Tome and Principe
○ Saudi Arabia
○ Senegal
○ Serbia
○ Seychelles
○ Sierra Leone
○ Singapore
○ Sint Maarten
○ Slovakia
○ Slovenia
○ Solomon Islands
○ Somalia
○ South Africa
○ Spain
○ Sri Lanka
○ Sudan
○ Sudan, South
○ Suriname
○ Swaziland
○ Sweden
○ Switzerland
○ Syria
○ Taiwan
○ Tajikistan
○ Tanzania
○ Thailand
○ Togo
○ Tonga
○ Trinidad and Tobago
○ Tunisia
○ Turkey
○ Turkmenistan
○ Tuvalu
○ Uganda
○ Ukraine
○ United Arab Emirates
○ United Kingdom
○ United States
○ Uruguay
○ Uzbekistan
○ Vanuatu
○ Vatican City
○ Venezuela
○ Vietnam
○ Virgin Islands, British
○ Virgin Islands, U.S.
○ Yemen
○ Zambia
○ Zimbabwe

2 Your gender:

○ Female
○ Male

3 Your age:

○ Less than 30
○ 30-39
○ 40-49
○ 50-59
○ 60-69
○ Greater than 69

4 Your specialty (Please choose all that apply):

☐ Allergy/Immunology
☐ Dermatology
☐ Paediatrics
☐ General practice
☐ Other: ______________________

5 Length of time in your current field of practice (years):

◯ Less than 5 years
◯ Between 5 and 9 years
◯ Between 10 and 14 years
◯ Between 15 and 19 years
◯ Over 19

6 You work at (Please choose all that apply):

☐ Private practice
☐ University clinic
☐ Hospital
☐ Specialized urticaria centre
☐ Other: ______________________

7 You primarily see:

◯ Outpatients
◯ Inpatients
◯ Other

8 You primarily see:

◯ Adults
◯ Children
◯ Both

9 How many Chronic Spontaneous Urticaria (CSU) patients do you see per week?

◯ 0-5
◯ 6-9
◯ 10-20
◯ 21-30
◯ 31-40
◯ 41-50
◯ >50
◯ Other (please specify)

10 How often do your CSU patients complain of angioedema? (% from the total number of patients)

◯ Less than 10%
◯ 10-20%
◯ 21-30%
◯ 31-40%
◯ 41-50%

◯ 51-60%
◯ 61-70%
◯ 71-80%
◯ 81-90%
◯ Over 90%

11 Please specify the urticaria guidelines you are aware of (Please choose **all** that apply):

☐ EAACI/WAO/GA2LEN/EDF
☐ AAAAI practice parameter
☐ National guidelines
☐ I am not aware of any
☐ Other: ______________________

12 Which of the guidelines do you follow in clinical practice (Please choose **all** that apply):

☐ EAACI/WAO/GA2LEN/EDF
☐ AAAAI practice parameter
☐ National guidelines
☐ I don’t use any
☐ Other: ______________________

13 If you DO NOT FOLLOW OR DEVIATE from the guidelines, please indicate the reasons behind it (Please choose **all** that apply):

☐ The guidelines do not undergo revision frequently enough
☐ I rely more on my own clinical experience
☐ I do not agree with the guidelines' recommendations and/or conclusions
☐ Some of the recommendations are unclear to me and require further detalization
☐ Some of the guidelines' recommendations cannot be implicated in my country of residence
☐ I had a negative experience with following the guidelines in my clinical practice
☐ Overly simplified approach to CSU management recommended by the guidelines that does not reflect the complexity of the disease
☐ The discrepancy and/or disagreement between the guidelines
☐ Not applicable
☐ Other: ________________________

14 Which of the following tests do you order or perform <u>in all CSU patients</u>? (Please choose **all** that apply):

☐ Complete blood count (CBC) with differential
☐ Erythrocyte sedimentation rate (ESR)
☐ C-reactive protein (CRP)
☐ Anti-thyroid antibodies (anti-TG/TPO)
☐ Thyroid stimulating hormone (TSH)

☐ Total IgE
☐ Eosinophil cationic protein (ECP)
☐ D-dimer
☐ Skin prick tests
☐ Allergen-specific IgE
☐ Antinuclear antibodies (ANA)
☐ Tryptase
☐ Autologous serum skin test (ASST)
☐ Search for chronic infections
☐ None
☐ Other: ________________________

15 Which of the following tests do you order or perform in patients with long-standing (>6 months), severe(symptoms interfering with normal daily activity and sleep) and poorly-controlled disease (lack of response to antihistamines)? (Check any that apply in addition to those selected in previous question):

☐ CBC with differential
☐ ESR
☐ CRP
☐ Anti-thyroid antibodies (anti-TG/TPO)
☐ TSH
☐ Total IgE
☐ ECP
☐ D-dimer
☐ Skin prick tests
☐ Allergen-specific IgE
☐ ANA
☐ Tryptase
☐ ASST
☐ Search for chronic infections
☐ None
☐ Other: __________________________

16 How often do you elucidate a cause of CSU, %? (Note: the cause of CSU is the condition that when treated leads to improvement or resolution of CSU):

○ Less than 10%
○ 10-20%
○ 21-30%
○ 31-40%
○ 41-50%
○ 51-60%
○ 61-70%
○ 71-80%
○ 81-90%
○ Over 90%

17 What are the common etiologies of CSU in your patients? (Please rank the frequency of CSU causes from 1 to 8: the most common cause - 1, the least common - 8)

☐ Idiopathic
☐ Type-I-Allergy
☐ Autoimmunity/autoreactivity
☐ Underlying systemic disorders
☐ Malignancy
☐ Chronic infections
☐ Food intolerance
☐ Non-neoplasm underlying systemic disorder
☐ Others

18 Which of the following medications do you administer a CSU patient as a first-line treatment? (Multiple answers will be regarded as a combination of medications used in the same patient)**:**

☐ First-generation H1-antihistamines
☐ Second-generation H1-antihistamines at standard dose
☐ Updosed second-generation H1-antihistamines (2-4 times the standard dose)
☐ H2-antihistamines (e.g. famotidine or ranitidine)
☐ Ciclosporin
☐ Omalizumab
☐ Montelukast
☐ Dapsone
☐ Systemic corticosteroids (for less than 10 days)
☐ Systemic corticosteroids (for more than 10 days in a row)
☐ Other: ___________________________

19 Which of the following medications do you administer a CSU patient as a second-line treatment? (Multiple answers will be regarded as a combination of medications used in the same patient. Answer this question independently from question #18)***:***

☐ First-generation H1-antihistamines
☐ Second-generation H1-antihistamines at standard dose
☐ Updosed second-generation H1-antihistamines (2-4 times the standard dose)
☐ H2-antihistamines (e.g. famotidine or ranitidine)
☐ Ciclosporin
☐ Omalizumab
☐ Montelukast
☐ Dapsone
☐ Systemic corticosteroids (for less than 10 days)
☐ Systemic corticosteroids (for more than 10 days in a row)
☐ Other: ___________________

20 Which of the following medications do you administer a CSU patient as a third-line treatment? (Multiple answers will be regarded as a combination of medications used in the same patient. Answer this question independently from questions #18, 19):

☐ First-generation H1-antihistamines
☐ Second-generation H1-antihistamines at standard dose
☐ Updosed second-generation H1-antihistamines (2-4 times the standard dose)
☐ H2-antihistamines (e.g. famotidine or ranitidine)
☐ Ciclosporin
☐ Omalizumab
☐ Montelukast
☐ Dapsone
☐ Systemic corticosteroids (for less than 10 days)
☐ Systemic corticosteroids (for more than 10 days in a row)
☐ Other: ____________________

21 Please indicate your opinion on the overall efficacy of the following agents in treatment of CSU. Please choose the appropriate response for each item:

	**Very high**	**High**	**Moderate**	**Low**	**Not effective**
H1-antihistamines of first generation	○	○	○	○	○
H1-antihistamines of second generation in standard dose	○	○	○	○	○
H1-antihistamines of second generation in high doses (2-4 times higher)	○	○	○	○	○
H2-antihistamines (e.g. famotidine or ranitidine)	○	○	○	○	○
Ciclosporin	○	○	○	○	○
Omalizumab	○	○	○	○	○
Montelukast	○	○	○	○	○
Dapsone	○	○	○	○	○
Systemic corticosteroids (for less than	○	○	○	○	○

	Very high	High	Moderate	Low	Not effective
10 days)					
Systemic corticosteroids (for more than 10 days in a row)	○	○	○	○	○

22 Please assess tolerability of the following agents in treatment of CSU. Please choose the appropriate response for each item:

	Good	Moderate	Bad
H1-antihistamines of first generation	○	○	○
H1-antihistamines of second generation in standard dose	○	○	○
H1-antihistamines of second generation in high doses (2-4 times higher)	○	○	○
H2-antihistamines (e.g. famotidine or ranitidine)	○	○	○
Ciclosporin	○	○	○
Omalizumab	○	○	○
Montelukast	○	○	○
Dapsone	○	○	○
Systemic corticosteroids (for less than 10 days)	○	○	○
Systemic corticosteroids (for more than 10 days in a row)	○	○	○

23 Do you record data from CSU patients?

- ◯ No
- ◯ Yes - Own database
- ◯ Yes - Hospital/Intitution's database
- ◯ Yes - National registry
- ◯ Yes - Registry organized by a Allergy or Dermatology medical society
- ◯ Yes - Other

24 Have you ever heard of the possibility to enter patient data into the chronic urticaria registry (CURE) for scientific analyses (www.urticaria-registry.com)?

Please choose **only one** of the following:

- ◯ Yes
- ◯ No

Thank you for taking your time to participate in our survey! If there are any comments or suggestions please enter them below.

Please write your answer here:

____

Thank you very much for your participation!
